# Collembola interact with mycorrhizal fungi in modifying oak morphology, C and N incorporation and transcriptomics

**DOI:** 10.1098/rsos.181869

**Published:** 2019-03-06

**Authors:** Marcel Graf, Markus Bönn, Lasse Feldhahn, Florence Kurth, Thorsten E. E. Grams, Sylvie Herrmann, Mika Tarkka, Francois Buscot, Stefan Scheu

**Affiliations:** 1 J.F. Blumenbach Institute of Zoology and Anthropology, University of Göttingen, 37073 Göttingen, Germany; 2 Department of Soil Ecology, Helmholtz Center for Environmental Research – UFZ Halle, 06120 Halle/Saale, Germany; 3 Department of Ecology and Ecosystem Science, Plant Ecophysiology, Technische Universität München, 85345 Freising, Germany; 4 German Centre for Integrative Biodiversity Research (iDiv) Halle-Jena-Leipzig, 04103 Leipzig, Germany; 5 Centre of Biodiversity and Sustainable Land Use, University of Göttingen, 37075 Göttingen, Germany

**Keywords:** Collembola, *Quercus robur*, plant nutrition, stable isotopes, plant defence, Gene Ontology

## Abstract

Soil detritivores such as Collembola impact plant growth, tissue nutrient concentration and gene expression. Using a model system with pedunculate oak (*Quercus robur*) microcuttings that display a typical endogenous rhythmic growth with alternating shoot (SF) and root flushes (RF), we investigated the transcriptomic response of oak with and without mycorrhiza (*Piloderma croceum*) to the presence of Collembola (*Protaphorura armata*), and linked it to changes in resource allocation by pulse labelling the plants with ^13^C and ^15^N. Collembola impacted Gene Ontology (GO) terms as well as plant morphology and elemental ratios with the effects varying markedly with developmental phases. During SF Collembola increased GO terms related to primary growth and this was mirrored in increased ^13^C and ^15^N excess in aboveground plant compartments. During RF, Collembola increased GO terms related to plant secondary metabolism and physical fortification. Further, Collembola presence resulted in an increase in plant defence-related GO terms suggesting that Collembola in the rhizosphere prime oak shoots against the attack by fungi or herbivores. Notably, the impact of Collembola on growth, resource allocation and oak gene expression was modified by presence of *P. croceum*. The results indicate that oaks clearly react to the presence of Collembola in the rhizosphere and respond in a complex way by changing the expression of genes of both primary and secondary metabolism, and this resulted in concomitant changes in plant morphology and physiology.

## 1. Introduction

Detritivore animals form part of the biota responsible for the recycling of plant litter and therefore contribute to the provisioning of nutrients to plants. Thereby, they indirectly impact plant growth and plant performance. However, detritivores also modify plant growth by a number of other mechanisms including modifying soil structure, grazing on rhizosphere microorganisms, altering plant–mycorrhiza symbiosis and changing root pathogen infections [1–3].

The great majority of plants are colonized by mycorrhizal fungi [4] which, by exploiting nutrients in the rhizosphere via extra-radical hyphae, foster the uptake of nutrients. Via their hyphal network, mycorrhizal fungi are embedded into the belowground food web and interact with detritivore animals. Grazing by fungal feeding soil invertebrates may detrimentally affect mycorrhizal fungi, but, on the other side, plants may benefit from nutrients made available, e.g. in animal excrements.

Collembola, as major soil detritivores reaching densities of more than 100 000 individuals per square metre [5], have been shown to graze on hyphae and mycelial mats of ectomycorrhizal fungi thereby impacting plant–mycorrhiza interrelationships [6,7]. Further, Collembola have been shown to increase the supply of nutrients to plants due to processing of detritus [8,9]. Overall, in the great majority of studies the presence of Collembola caused an increase in plant nutrient uptake and plant tissue nutrient concentration, and thereby fostered plant growth and performance [10–15], suggesting that the Collembola-mediated increase in nutrient mobilization outweighs their detrimental effect via grazing on mycorrhizal fungi. In fact, there is evidence that moderate grazing on fungal hyphae by fungal feeding soil invertebrates increases fungal productivity and functionality due to liberating nutrients from senescent hyphae thereby fostering fungal regrowth [16,17]. Further, there is evidence that Collembola preferentially graze on saprotrophic rather than mycorrhizal fungi [2,9], thereby favouring the competitive strength of mycorrhiza, mycorrhizal nutrient capture and plant growth. However, non-nutrient effects may also contribute to Collembola-mediated changes in mycorrhizal functioning and plant growth. For example, Collembola have been shown to alter root morphology without changing plant nutrient concentrations [18,19]. To disentangle nutrient- and non-nutrient-based effects of Collembola on plant growth and elucidate the role of mycorrhiza in Collembola–plant interactions, laboratory experiments are needed, manipulating mycorrhiza and Collembola independently and investigating the transcriptional response of plants to these manipulations. Further, for understanding plant responses, detailed analysis of plant growth as well as plant carbon and nutrient allocation are needed. Unfortunately, experiments combining these approaches are lacking. The only study investigating the transcriptional response of plants to the presence of Collembola focused on *Arabidopsis thaliana*, a non-mycorrhizal plant species [20]. The study showed that the presence of Collembola modified gene expression with both genes of primary and secondary metabolic pathways being altered. Other studies using earthworms and dung beetles also showed that detritivore–plant interactions change plant secondary metabolite synthesis and defence characteristics of plants against herbivores [21–24]. However, only herbaceous plants have been considered in these studies, while detritivore-mediated changes in gene expression patterns in trees have not been studied until today. This is surprising because detritivore animals reach maximum density in forests and therefore are likely to interact with trees and the mycorrhizal fungi with which they are associated [14].

To disentangle detritivore–mycorrhiza interactions and their effects on the transcriptional and growth response of trees we established an experimental model system including an ectomycorrhizal tree, *Quercus robur* (L.), the ectomycorrhizal fungal species *Piloderma croceum* (J. Erikss. & Hjortst. Strain 729; DSM-4924) and the Collembola species *Protaphorura armata* (Tullberg)*. Quercus robur* is among the most common European tree species and of significant economic importance. *Piloderma croceum* is a widespread ectomycorrhizal fungal species in deciduous forests and *P. armata* is a widespread soil living (euedaphic) Collembola species common in the rhizosphere of plants. To unravel morphological, nutritional and gene expression changes in *Q. robur* due to the presence of *P. croceum* and *P. armata* we established a full factorial experiment varying the presence of mycorrhiza and Collembola. As oak displays rhythmic growth with alternating shoot (SF) and root flushes (RF) we investigated both of these growth phases (cf. [25]). The study formed part of the TrophinOak project (http://www.trophinoak.de) aiming at investigating in a comprehensive way the transcriptomic response of *Q. robur* to biological interactors including mutualists and antagonists [26–28]. Further, using ^13^C and ^15^N labelling, the influence of *P. armata* and *P. croceum* on carbon and nitrogen allocation during the rhythmic growth of *Q. robur* was investigated. As the response of *Q. robur* to the mycorrhizal fungus *P. croceum* has been investigated in detail [29,30], we focus on effects of *P. armata* and its interaction with *P. croceum* in the present study. We hypothesized that (i) gene expression patterns of oaks are altered in the presence of Collembola with the patterns varying between SF and RF, and including both genes involved in primary and secondary metabolism; (ii) these changes are attenuated in presence of mycorrhiza, assuming that both Collembola and mycorrhiza improve plant nutrient supply; (iii) Collembola-mediated changes in the expression of genes involved in plant primary metabolism reflect respective changes in plant carbon and nitrogen uptake, i.e. upregulation of genes related to plant growth are associated by increased incorporation of ^13^C and ^15^N; and (iv) Collembola-mediated changes in the expression of genes involved in plant secondary metabolism reflect upregulation of genes involved in plant defence against herbivores.

## 2. Material and methods

### 2.1. Quercus robur

The present study and other studies in the framework of the TrophinOak consortium [26,28–30] employed clonally propagated pedunculate oak microcuttings (DF159). Propagating and rooting of microcuttings was performed at the Helmholtz Center for Environmental Research (UFZ; Halle, Germany) as described previously [25,31]. The root system of single microcuttings was implanted into Petri dishes (12 × 12 cm) filled with sterilized soil with the shoots growing outside the microcosms. The soil was taken from an oak forest stand at the Dölauer Heide close to Halle/Saale, Saxony Anhalt, Germany (51.51016° N, 11.91291° E). The O_h_ and upper A_h_ layers of the soil were taken, homogeneously mixed by sieving (5 mm mesh), air-dried, mixed with sand (1 : 1, v/v), packed in 500 ml aliquots and sent for γ-radiation for sterilization (50 kGy; Beta-Gamma-Service, Wiehe, Germany). The soil/sand mixture contained 4.88 ± 0.11% carbon, had a C-to-N ratio of 19.3 and a pH of 4.12. Sterilized soil aliquots were stored at 8°C and sterility was tested before use by plating on Lysogeny broth agar.

The experiment was set up in a climate chamber at 23°C, relative air humidity of 75% and a photosynthetic photon flux density of 180 µmol m^−2^ s^−1^ at long day conditions (16/8 h). A total of 129 oak microcuttings were used, which under the specified conditions establish an endogenous rhythmic growth as described in Herrmann *et al.* [25]. High relative humidity reduces plant stress, but does not improve mycorrhization of oak microcuttings [21]. However, although not fully mycorrhized, oak microcuttings treated in that way show typical effects of mycorrhizal plants, i.e. increased growth, photosynthesis and stress resistance [11,28,29,31].

### 2.2. Piloderma croceum

The ectomycorrhizal fungus *P. croceum* (*Piloderma*) was reared and cultivated at 20°C in darkness in Petri dishes containing Modified Melin-Norkrans agar (for details see [25]). *Piloderma croceum* is a widespread mycorrhizal fungus preferentially colonizing deciduous trees such as pedunculated oak [25,31]. Microcosms were inoculated with *Piloderma* as described in Tarkka *et al.* [28]. In brief, the *Piloderma* inoculum was produced using a substrate mixture of vermiculite (675 ml), sphagnum peat (75 ml), and Melin-Norkrans liquid medium (300 ml) modified by Marx [32] without carbohydrates and with 1/10 strength for phosphorus and nitrogen as described in Herrmann *et al.* [25] using a 48-day-old liquid fungal culture reared in 100 ml glass flasks at 20°C in darkness with agitation (100 r.p.m.). The inoculum was incubated in darkness at 20°C for four weeks and used for mycorrhiza establishment. Mycorrhization was checked after five weeks using a dissecting microscope and showed that the inoculation was successful.

### 2.3. Protaphorura armata

The Collembola species *P. armata* (*Protaphorura*) was taken from laboratory cultures established from field populations close to Darmstadt (Germany) in 2002. *Protaphorura armata* is widespread in Europe and preferentially colonizes the mineral soil and rhizosphere of plants (euedaphic species). Cultures were kept on a mixture of sterilized potting soil and clay pellets (3 : 1) at 14°C in darkness and fed with moistened baker's yeast. Collembola treatments received 90 *P. armata* individuals which were added six weeks after transplantation of microcuttings into the microcosms. Collembola were added after the mycorrhizal inoculum to allow mycorrhizal fungi to establish without being grazed, i.e. to ensure effective mycorrhization of the microcuttings.

### 2.4. ^13^C and ^15^N labelling

Twenty-four hours before harvest, plants were transferred into a plexiglas chamber and labelled using a mobile ^13^CO_2_ labelling system controlling CO_2_ concentration and air humidity (set to 400 µl l^−1^ and 70%, respectively) in the chamber (see [26,31]). CO_2_ in ambient air was removed and replaced by CO_2_ containing 8.3 ± 0.2 atom% ^13^CO_2_ (mean ± s.d.) (Eurisotop, Saarbrücken, Germany). Thereby, *Q. robur* microcuttings were exposed to the ^13^CO_2_-enriched atmosphere over a complete light period of 16 h and the CO_2_ concentration adjusted to 400 ± 2 ml l^−1^ as described in Herrmann *et al.* [30]. ^15^N labelling was performed 72 h before harvest using 98 atom% ^15^NH_4_^15^NO_3_ (Sigma, Darmstadt, Germany); 0.1 mg ^15^NH_4_^15^NO_3_ dissolved in 5 ml sterile distilled water was injected into the rhizosphere under sterile conditions.

### 2.5. Experimental procedure

The experiment was set up in a full factorial design with the factors *Piloderma* (with and without) and *Protaphorura* (with and without) allowing to inspect effects of individual organisms but also their interaction. Microcosms were incubated in a climate chamber at the conditions specified above for 8 weeks and then destructively sampled. During incubation the position of individual microcosms was changed in a random way twice a week. Depending on the rhythmic growth phase of microcuttings at harvest, individual experimental systems were grouped into SF and RF. The grouping was based on the opening of individual buds and the anthocyane colouring of leaves as described in more detail in Herrmann *et al.* [25]*.* Microcosms with microcuttings which could not be ascribed unequivocally to either of these phases were discarded.

From the initial 129 microcuttings a total of 78 reached the root or shoot flush stage at harvest. Of those, 49 microcuttings were assigned to RF and 29 to SF, the remaining microcuttings were discarded. As defined in Herrmann *et al*. [25] RF microcuttings are characterized by rush bud stage B which is associated by maximum root growth, and SF microcuttings are characterized by stage D which is associated by maximum leaf expansion. Petri dishes of the 78 microcosms were opened and microcuttings were purged from soil. Harvest started early in the morning (08.00) and lasted until the afternoon (17.00). Microcosms were processed sequentially in random order with handling of individual microcosms and placement of plant tissue into N_2_ lasting a maximum of 5 min. Microcuttings were cut into five compartments: source leaves (i.e. fully expanded leaves with net export of photosynthates to growing parts of the plant), sink leaves (i.e. not fully expanded leaves with proliferating cells during plant growth), stem, principal root and lateral roots. The principal root comprised the most developed root from which lateral roots branched. The compartments were weighed and the length of stems was measured. Based on total plant biomass at harvest and duration of incubation the relative growth rate of microcuttings was calculated assuming linear growth. Then, the compartments were wrapped into aluminium foil, frozen in liquid nitrogen and stored at −80°C. Some plant compartments (leaves and lateral roots) were pooled before ^13^C and ^15^N analysis and RNA extraction to obtain enough material for the analyses. Each pool included 2–5 individual plant compartments (see electronic supplementary material, table S2).

### 2.6. RNA assay

Transcriptome analysis was based on pooled leaf samples; a total of 19 samples were used (see electronic supplementary material, table S2). RNA extraction was performed using the MasterPure Plant RNA Purification Kit (Epicentre Technologies Corporation, Madison, WI, USA). According to the manufacturer protocol 50 mg of leaf tissue were used for RNA extraction. Unfortunately, there was not enough tissue material to also perform transcriptome analysis of roots. Quality controls were conducted using formaldehyde-agarose gels, Nanodrop spectrophotometer (Thermo Scientific, Waltham, MA, USA) and Bioanalyzer 2100 (Agilent, Santa Clara, CA, USA). The extracted RNA was used to accomplish 100 bp paired-end libraries. RNA was sequenced using an Illumina HiSeq 2000 at Beijing Genomics Institute, Hong Kong, China.

### 2.7. Read processing and analysis of differential gene expression

Reads were processed following Tarkka *et al.* [28]. Briefly, low-quality sequences and sequencing artefacts were removed with SeqClean (http://sourceforge.net/projects/seqclean/files/) and low-quality sequencing ends were trimmed with a custom Java script. Short sequences (less than 50 bp) and sequences lacking paired-end information were discarded. The processed Illumina reads were aligned against the reference transcriptome OakContigDF159.1 [28] by Bowtie [33] and quantified by RSEM [34]. The mapping rate onto the OakContigDF159 reference was 76.5–80.4%. Fold changes in gene expression were calculated by pairwise comparisons using the edgeR function [35] implemented in the Bioconductor package [36]. In these comparisons, negative binomial models are fitted to the transcript abundancies determined by RSEM. Contigs were considered differentially expressed when the Benjamini–Hochberg adjusted *p*-value of this fit was less than 1%. Blast2GO was used to get a description for each contig based on up to 20 hits against NCBI NR database in a blastx search (*E*-value 1 × 10^−5^). Protein sequences from *Arabidopsis thaliana* TAIR database were downloaded to perform a blastx search of DF159.1 and to assign homologue proteins from *A. thaliana* to each contig. Only hits with an *E*-value of at least 1 × 10^−5^ were taken into account. The best *A. thaliana* protein hit for each oak contig was determined by taking the *A. thaliana* protein exhibiting the largest per cent identity to the contig in the local alignment. Gene Ontology (GO) [37] enrichment analysis was performed with the Bioconductor package GOseq [38]. GOseq performs a statistical test based on a hypergeometric distribution to determine if in a given list of DE tags (e.g. genes or contigs) tags assigned to a certain category (e.g. GO terms) are significantly enriched, i.e. if they occur more frequently than expected by chance. Thereby, GOseq adjusts the estimation of the *p*-value for tag-length. We used the capability of GOseq to perform enrichment analyses for a second type of categories, protein families (Pfam). The OakContigDF159.1 reference library, GO annotations as well as best blast hits of each contig have been deposited at www.trophinoak.de. This publication will focus on results of treatments containing Collembola; straight *Piloderma* effects are described in Herrmann *et al.* [29].

### 2.8. Carbon and nitrogen allocation analysis

For ^13^C and ^15^N analysis of the compartments of oak microcuttings, the plant material was dried, milled, weighed into tin capsules (1.50–1.75 mg) and stored in a desiccator until analysis (plant material contained pools (leaves and lateral roots) or individual plants (stems and principal roots); for pools see electronic supplementary material, table S2). For ^13^C and ^15^N analysis of Collembola, 15–20 individuals of *P. armata*, equivalent to 50–200 µg dry weight, were transferred into tin capsules, dried at 60°C for 24 h and stored in a desiccator until analysis.

Samples were analysed with a combined system consisting of an elemental analyser (NA 1500, Carlo Erba, Milan, Italy) and a mass spectrometer (MAT 251, Finnigan, Bremen, Germany) [39]. The precision of the measurement is 0.1 delta per mil for ^13^C and 0.2 delta per mil for ^15^N. Stable isotope abundance is expressed as atom% excess calculated as ^13^C (%) = [^13^C/(^12^C + ^13^C)]/100 and ^15^N (%) = [^15^N/(^14^N + ^15^N)]/100. For ^13^C PD belemnite (PDB) and for ^15^N atmospheric N was used as primary standard. Acetanilide (C_8_H_9_NO; Merck, Darmstadt, Germany) was used for internal calibration.

### 2.9. Statistical analysis

**Figure 1.**
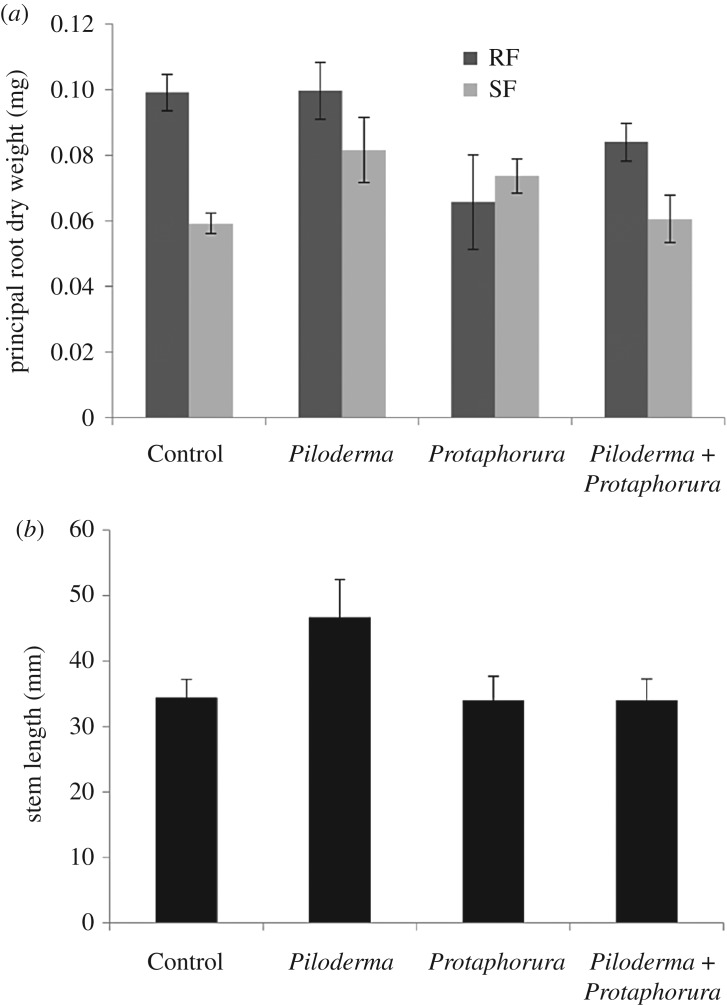
Effects of *Piloderma*, *Protaphorura* and Stage on (*a*) principal root dry weight during root (RF) and shoot flush (SF) (Control *n* = 9, *Piloderma n* = 15, *Protaphorura n* = 6 and *Protaphorura* + *Piloderma n* = 20 in RF, and Control *n* = 9, *Piloderma n* = 7, *Protaphorura n* = 6 and *Protaphorura* + *Piloderma n* = 5 in SF treatments), and (*b*) on stem length (pooled for RF and SF) (Control *n* = 18, *Piloderma n* = 22, *Protaphorura n* = 12 and *Protaphorura* + *Piloderma n* = 25).

**Figure 2.**
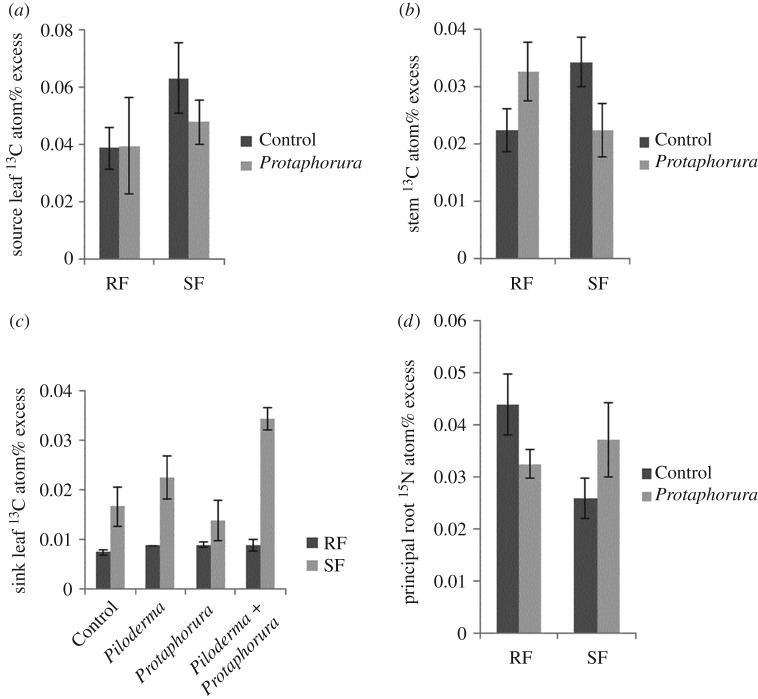
Effects of (*a*) *Protaphorura* on source leaf ^13^C atom% excess during root (RF) and shoot flush (SF) (pooled for *Piloderma*) (RF: Control *n* = 6; *Protaphorura n* = 4; SF: Control *n* = 5; *Protaphorura n* = 6); ^13^C and ^15^N atom% excess in plant compartments, (*b*) effects of *Protaphorura* on stem ^13^C atom% excess during RF and SF (pooled for *Piloderma*) (RF: Control *n* = 6, *Protaphorura n* = 4; SF: Control *n* = 5, *Protaphorura n* = 4), (*c*) effects of *Protaphorura* and *Piloderma* and their combination on sink leaf ^13^C atom% excess during RF and SF (RF: Control *n* = 5, *Piloderma n* = 6, *Protaphorura n* = 3, *Protaphorura* + *Piloderma n* = 6; SF: Control *n* = 5, *Piloderma n* = 3, *Protaphorura n* = 3, *Protaphorura* + *Piloderma n* = 6), and (*d*) effects of *Protaphorura* on principal root ^15^N atom% excess (pooled for *Piloderma*) (RF: Control *n* = 6, *Protaphorura n* = 3; SF: Control *n* = 5, *Protaphorura n* = 3).

^13^C and ^15^N values of plant tissue (i.e. lateral root, principal root, sink leaf, source leaf, stem) as well as plant biomass, relative growth rate and stem length were analysed using three-factorial general linear model (GLM), accounting for the non-balanced design, with the factors Stage (RF and SF), *Protaphorura* (with and without) and *Piloderma* (with and without). Significance level was set to *p* < 0.05. Statistical analyses were carried out using SAS (SAS Institute Inc., Cary, NC, USA). As not all plant compartments could be analysed because of lack of enough material in each of the treatments, the number of replicates of the individual variables analysed differed and is given in the legends of figures 1 and 2.

## 3. Results

### 3.1. Morphology

*Protaphorura* significantly affected principal root biomass, but the effect varied with growth stage of the microcuttings and presence of *Piloderma* (significant *Protaphorura* × Stage × *Piloderma* interaction; *F*_1,70_ = 4.01, *p* = 0.0492; figure 1*a*; for complete results of statistical analyses see electronic supplementary material, table S1). *Protaphorura* reduced principal root biomass during RF, with the effect being less pronounced in presence of *Piloderma*. In contrast, during SF *Protaphorura* increased principal root biomass, with the effect being less pronounced in presence of *Piloderma*. Additionally, presence of *Protaphorura* significantly affected stem length, but the effect varied with the presence of *Piloderma* (significant *Protaphorura* × *Piloderma* interaction; *F*_1,70_ = 4.13, *p* = 0.046; figure 1*b*). *Protaphorura* reduced stem length during RF, but the effect was less pronounced in presence of *Piloderma*. In contrast, during SF *Protaphorura* increased stem length, but again the effect was less pronounced in presence of *Piloderma*. Further, the presence of *Protaphorura* uniformly reduced relative growth rate independent of stage and presence of *Piloderma* by on average 9.8% (*F*_1,53_ = 5.81, *p* = 0.019).

### 3.2. ^13^C and ^15^N uptake

The presence of *Protaphorura* significantly affected source leaf ^13^C atom% excess, but the effect varied with growth stage of the microcuttings (significant *Protaphorura* × Stage interaction; *F*_1,30_ = 4.82, *p* = 0.036; figure 2*a*); during RF *Protaphorura* slightly increased source leaf ^13^C atom% excess, whereas during SF it was strongly reduced. Further, *Protaphorura* significantly affected stem ^13^C atom% excess, but the effect varied with stage (significant *Protaphorura* × Stage interaction; *F*_1,31_ = 7.58, *p* = 0.010; figure 2*b*); during RF *Protaphorura* increased stem ^13^C atom%, whereas during SF it was decreased. Furthermore, sink leaf ^13^C atom% excess was increased by *Protaphorura*, but the effect varied with the presence of *Piloderma* and with stage (significant *Protaphorura* × *Piloderma* × Stage interaction; *F*_1,26_ = 4.83, *p* = 0.037; figure 2*c*); the effect was restricted to SF and was most pronounced in presence of both *Protaphorura* and *Piloderma.*

In addition to aboveground plant compartments, *Protaphorura* significantly affected ^15^N atom% excess of principal roots, but again the effect varied with stage (significant *Protaphorura* × Stage interaction; *F*_1,29_ = 4.73, *p* = 0.038; figure 2*d*). *Protaphorura* reduced principal root ^15^N atom% excess during RF, but increased it during SF. Independent of the presence of *Protaphorura*, stem ^15^N atom% excess was increased during RF as compared to SF by 63% (*F*_1,31_ = 9.17, *p* = 0.005). Similarly, in lateral roots, ^15^N atom% excess was increased during RF as compared to SF by 27% (*F*_1,22_ = 7.42, *p* = 0.012) (data not shown).

### 3.3. Differential expression profiles

In the *Protaphorura* treatment 467 and 120 contigs were differentially expressed in leaves during SF and RF, respectively. Of the differentially expressed contigs during SF, 410 were enriched in upregulated and 57 were enriched in downregulated contigs; respective numbers during RF were 65 and 55. In the combined treatment with *Protaphorura* and *Piloderma*, 1004 and 104 contigs were differentially expressed in leaves during SF and RF, respectively. Of the differentially expressed contigs during SF, 591 were enriched in upregulated and 413 in downregulated contigs; respective numbers during RF were 46 and 58.

**Figure 3.**
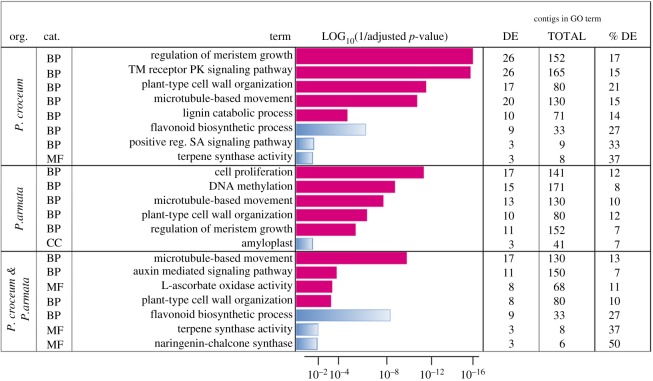
Results of Gene Ontology terms over-representation analysis (FDR < 0.01). Gene Ontology (GO) terms enriched in up- and down-regulated contigs from sink leaves at shoot flush (SF) of plants treated with *Piloderma croceum*, *Protaphorura armata*, or from combined treatment are shown, with GO terms related to upregulated (red) and downregulated (blue) contigs. BP marks GO category biological process, MF molecular function and CC cellular compartment, and significance levels are marked by column lengths with maximum column length at *p* = 1 × 10^−16^. The absolute numbers and percentages of differentially expressed contigs under each GO term are indicated. TM, transmembrane; PK, protein kinase; SA, salicylic acid. Note that plant growth-related GO terms *regulation of meristem growth* and *microtubule based movement* are enriched in upregulated contigs at all three interaction types.

A number of enriched GO terms mirrored changes in oak gene expression levels in sink leaves during SF, and this was true for each pairwise comparison, i.e. Control versus *Piloderma*, Control versus *Protaphorura* and Control versus Combined (figure 3; electronic supplementary material, table S3). In each of the three treatments, the GO terms *microtubule-based movement* and *plant-type cell wall organization* were enriched in upregulated genes, as was the GO term *regulation of meristem growth* in Control versus *Piloderma* and Control versus *Protaphorura* treatments. By contrast, the term *flavonoid biosynthesis* was enriched in downregulated genes in the treatments Control versus *Piloderma* and Control versus Combined. Apart from this, the treatments showed specific patterns of GO enrichment. In presence of *Piloderma*, mainly growth- and development-related GO terms were enriched in upregulated contigs, as well as GO terms *transmembrane receptor protein tyrosine kinase signaling pathway* and *lignin catabolism*, whereas GO terms *salicylic acid mediated signaling pathway* and *terpene synthase activity* were enriched in downregulated contigs. In presence of *Protaphorura*, GO terms of *cell proliferation* and *DNA methylation* were enriched in upregulated contigs during SF, but the GO term *amyloplasts* was enriched in downregulated contigs. In the combined treatment, enriched GO terms included *auxin mediated signaling pathway* and *L-ascorbate oxidase activity.* By contrast, the GO terms *oxidation-reduction process*, *terpene synthase activity* and *naringenin-chalcone synthase activity* were enriched in downregulated contigs. In the *Protaphorura* treatment, two defence-related contigs were enriched in upregulated contigs during SF in leaves. These included the *enhanced disease susceptibility 5* contig, which is connected to defence responses including *salicylic acid mediated pathway*, *jasmonic acid mediated pathway* and *response to chitin*, and a contig related to a *chalcone-flavanone isomerase family protein*, which forms part of the flavonoid biosynthesis (table 1). In the combined treatment with *Protaphorura* and *Piloderma*, no enriched contigs related to defence were present. Generally, gene-expression in the combined treatment was least diverse.

**Table 1.** Defence- and secondary-metabolism-related contigs with consistent enrichment or depletion. Pairwise comparisons Control-Pilo (*Piloderma* treatment), Control-Prot (*Protaphorura* treatment) and Control-Combined (Combined treatment) in leaves of oak microcuttings during two developmental stages, i.e. shoot flush (SF) and root flush (RF). Significant enrichment (orange) was determined by edgeR with a threshold Benjamini-corrected *p*-value of 0.01, indicated by FDR.

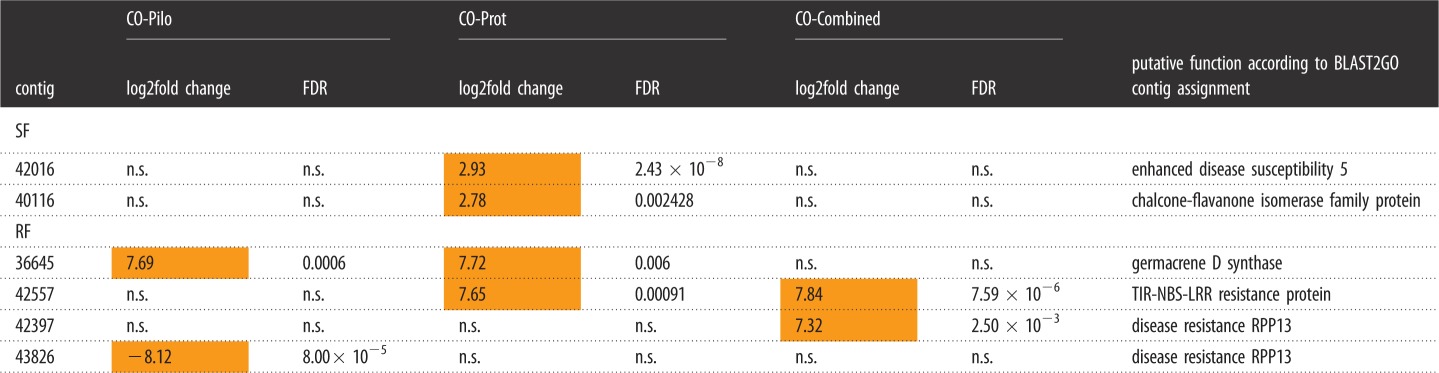

Only a few GO terms were enriched in source leaves during RF (electronic supplementary material, table S3). In presence of *Protaphorura*, GO terms *L-ascorbate oxidase activity*, *laccase activity* and *secondary cell wall biogenesis* were enriched in upregulated contigs. During RF, *acid phosphatase* transcripts were upregulated in all three treatments, *germacrene D synthase* by *Piloderma* and *Protaphorura*, and *TIR-NBS-LRR resistance protein* by *Protaphorura* and combined treatment. Further, there was a highly upregulated contig in presence of *Protaphorura* which potentially is related to defence, *disease resistance RPP13-like protein 1*, and interestingly, the abundance of a closely related transcript was downregulated by *Piloderma* (table 1)*.* Related to phenylpropanoid metabolism, *hydroxycinnamoyl-coenzyme A* transcripts were upregulated by *Piloderma*, and according to GO enrichment analysis, cell-wall-related transcripts were upregulated by *Protaphorura* encoding two laccases, cellulose synthases, a beta-1,4-xylosyltransferase and a proline-rich cell wall protein.

## 4. Discussion

Collembola are among the most widespread and abundant soil arthropods modifying plant performance in a multitude of ways. Modifications in plant performance at least in part are assumed to be due to Collembola interacting with rhizosphere microorganisms, in particular fungi including mycorrhiza [7,9,40,41]. The only study investigating changes in plant gene expression as affected by Collembola used *Arabidopsis thaliana* and showed that Collembola affected both plant primary and secondary metabolism [20]. Conforming to the finding that Collembola also affect plant secondary compounds, effects of Collembola on plant growth have been shown to propagate into the herbivore system affecting e.g. aphid reproduction [12,42]. The present study for the first time addressed the combined effect of Collembola and mycorrhizal fungi using a woody plant model system and targeted both changes in plant gene expression as well as plant carbon and nitrogen incorporation. As hypothesized, Collembola indeed altered plant gene expression, and impacted both plant carbon and nitrogen incorporation. Notably, this was true in presence and absence of mycorrhiza. Also, as hypothesized, the effect of Collembola on plant gene expression, and plant carbon and nutrient incorporation varied markedly between developmental stages of oaks, i.e. SF and RF.

We used *P. armata* as model Collembola species, which is widespread in forests but also agricultural systems in Europe. It represents euedaphic Collembola species which preferentially colonize the mineral soil and rhizosphere of plants. As Collembola are morphologically and trophically diverse and also colonize the soil surface (epedaphic species), their interaction with plants may be more diverse than indicated by investigating only a single species. However, representing euedaphic Collembola species, the effects caused by *P. armata* are likely to be typical for a wide range of Collembola species colonizing the rhizosphere of plants. *Piloderma croceum*, our model species of ectomycorrhizal fungi, is a broad host range ectomycorrhizal fungus and common mutualist of both conifer and hardwood species, typically occurring in boreal and temperate forests. The effects of *P. armata* and its interaction with *P. croceum*, as found with our model system, therefore are likely to be also of relevance in the field.

### 4.1. Shoot flush

*Protaphorura* significantly increased root biomass and also root ^15^N atom% excess during SF. Since oak roots are not growing during SF, roots mainly function in supplying aboveground plant compartments with nutrients and water. Supporting this scenario a GO term related to *water channel activity* was increased in each of the treatments during SF and this was most pronounced in the combined treatment with *Protaphorura* and *Piloderma*. This suggests that both Collembola and mycorrhiza fostered water and nutrient uptake in oak roots. Notably, this is in line with earlier findings that Collembola and mycorrhiza interact in fostering plant nitrogen uptake [14,19]. Additionally, in presence of *Protaphorura* the GO term *nutrient reservoir activity* was enriched in upregulated contigs in leaves, suggesting that *Protaphorura* stimulated the mobilization of nutrients from storage pools to support leaf development. Notably, the enrichment in the *nutrient reservoir activity* GO term was most pronounced in the combined treatment with *Protaphorura* and *Piloderma*, again suggesting that Collembola and mycorrhiza complement each other in fostering plant nutrition. Collembola-mediated changes in root growth and N incorporation are in line with earlier findings that Collembola alter root morphology [18,19] and nitrogen uptake by plants from soil organic matter [43,44]. The increased root biomass and root ^15^N atom% excess during SF in presence of *Protaphorura* and the observed increase in GO terms suggest that Collembola increased the availability of nutrients thereby triggering increased water and nitrogen uptake by oak microcuttings. This then triggered increased mobilization of nitrogen from plant reservoirs and transfer of both nitrogen taken up by plants and mobilized from plant reservoirs into sink leaves, thereby fostering plant growth.

In fact, during SF, presence of *Protaphorura* resulted in enrichment of upregulated contigs related to plant growth in leaves, such as *cell proliferation* and *regulation of cell cycle*; further, the *Piloderma*-mediated increase in ^13^C atom% excess in sink leafs during SF was more pronounced in presence of *Protaphorura*. Earlier studies also reported Collembola to increase plant growth [12,14,43,44]. Concomitant with the reduction in ^13^C atom% in source leaves and stems in presence of Collembola during SF, *Protaphorura* increased allocation of carbon resources from source to sink leaves thereby fostering leaf growth and expansion. Again, this supports the above scenario that the Collembola-mediated increase in plant nitrogen uptake triggered plant nutrient mobilization and plant growth.

Similar to leaves and stems, *Protaphorura* also affected carbon allocation to roots during SF. Complementary to the enrichment of upregulated contigs regarding the *nutrient reservoir activity* GO term, presence of *Protaphorura* led to enrichment of the GO term *amyloplast formation*, supporting the assumption that oak microcuttings increased investment in sink leaf development during SF. Further, *Protaphorura* reduced ^13^C atom% excess in source leaves and stems during SF, suggesting that carbon storage, predominantly occurring in source leaves, was reduced by *Protaphorura*. This indicates that the Collembola-mediated increase in the allocation of resources from roots to shoots fostered the investment of carbon by plants into growth rather than into storage. Supporting this conclusion, earlier studies showed that Collembola increase nitrogen concentration in plants and shift plant biomass towards shoots [43–45]. This again supports our conclusion that Collembola-mediated increase in plant nutrient concentrations triggers oak microcuttings to shift resource allocation from storage to growth via allocating carbon resources into actively growing sink leaves. Overall, the results suggest that Collembola-mediated changes in plant resource uptake and allocation as well as gene expression patterns are triggered by Collembola indirectly impacting plants via increasing nutrient availability, which probably resulted from Collembola grazing on rhizosphere microorganisms and thereby liberating nutrients bound in microbial biomass [46,47]. However, in addition to these nutrient-based effects, Collembola may also have triggered changes in plant performance via plants sensing chemical or mechanical cues of Collembola and modifying gene expression patterns [20,48,49].

In addition to genes related to plant primary metabolism, *Protaphorura* also altered the expression of defence-related contigs during SF, such as the *enhanced disease susceptibility 5* contig. This contig is related to the *salicylic acid mediated pathway*, *jasmonic acid mediated pathway* and *response to chitin*. Additionally, a defence-related contig related to *chalcone-flavanone isomerase family protein* forming part of flavonoid biosynthesis was enriched in upregulated contigs by *Protaphorura* during SF. Plant defence is known to be induced by herbivores including those feeding on roots [50,51]. However, induced defence has the disadvantage that plants may suffer from damage before the defence is in place. For preventing damage, plants may use environmental cues providing information on potential or upcoming attacks [52]. Due to the enrichment of upregulated contigs related to defence, *Protaphorura* probably primed oak seedlings against potential attacks by herbivores. Priming may be related to plants physically sensing the presence of rhizosphere arthropods, suggesting that discrimination of different arthropod taxa by plants is limited. Indeed, it has been shown that plants sensitively respond to mechanostimulation and alter gene expression patterns after contact [53,54]. Previous studies performed in the framework of the TrophinOak project demonstrated that the expression of defence genes also is elicited in SF by *Streptomyces* sp. and plant parasitic nematodes [27,55]. Similar to the present study, these responses were attenuated in presence of *Piloderma.* In the case of the bacterium, the priming-like response led to diminished powdery mildew symptoms. However, the response of oak microcuttings in the present study may also be based on *Protaphorura* feeding on plant roots, which occasionally occurs, but does not detrimentally affect plant growth [19]. Further, plants may also sense cues of the cuticle of Collembola, potentially chitin. In fact, the contig *enhanced disease susceptibility 5* is assumed to be related to the response of plants to chitin. Notably, sensing of other soil invertebrates than root herbivores may be advantageous as they may function as vectors for pathogenic microorganisms [56,57].

### 4.2. Root flush

In contrast to SF, *Protaphorura* significantly reduced ^15^N atom% excess of principal roots and reduced their biomass during RF. Biomass reduction of roots might be due to root feeding and in fact, *P. fimata*, a closely related species of *P. armata*, has been shown to switch diet from litter resources in soil to feeding on fine roots of *Zea mays* (L.) if available [58]. More intensive feeding on roots by Collembola during RF than during SF may reflect that during RF oaks are investing in root growth and therefore allocate plant resources, including compounds of high nutritional value such as sugars and amino acids, into roots.

Due to the low number of DE contigs, only a few GO terms in leaves were over-represented in source leaves at RF. Upregulated contigs related to *cell wall formation* suggests that during RF *Protaphorura* stimulated the establishment of mechanic barriers of plants and plant structural fortification, contrasting the stimulation of plant investment into growth during SF. Supporting this conclusion, presence of *Protaphorura* increased stem ^13^C atom% excess during RF. Similarly, Scheu *et al*. [59] showed that Collembola increase plant tissue C concentrations, suggesting that plant structural cell wall constituents were enhanced. Increased investment into structural components may explain why *Protaphorura* reduced shoot length during RF; notably, reduced shoot length in presence of Collembola has been reported previously [60]. Supporting the assumption that *Protaphorura* altered the investment of oaks into plant defence during RF, their presence led to an enrichment of contigs related to defence, namely *tir-nbs-lrr resistance protein* related to a wide spectrum of defence responses against antagonists. The above-mentioned reduction in root ^15^N concentrations and root biomass as well as the enrichment of a defence-related contig probably was due to *Protaphorura* feeding on roots (see above), resulting in increased defence-related GO terms.

The contrasting effects of *Protaphorura* during RF as compared to SF reflect the very different gene expression patterns of oak during these phases [29] and suggest that oaks very differently respond to environmental cues including interacting biota during the two allocation and developmental phases RF and SF. Further, during SF and RF *Protaphorura* were interacting with mycorrhiza regarding gene expression and nutrient uptake, with mycorrhiza attenuating the effect of *Protaphorura*. This is in line with our second hypothesis.

## 5. Conclusion

Collembola in the rhizosphere of oak seedlings significantly impacted plant gene expression patterns, plant morphology and plant carbon and nitrogen incorporation. Notably, effects of *Protaphorura* markedly varied during allocation phases, i.e. SF and RF. During both SF and RF, *Protaphorura* modified the expression of growth-specific genes which was mirrored in increased ^13^C and ^15^N plant tissue nutrient concentrations. In SF *Protaphorura* presence resulted in an enrichment of GO terms related to plant growth and plant primary metabolism. In contrast, during RF presence of *Protaphorura* led to an enrichment of GO terms related to physical plant fortification and plant secondary growth. In addition to plant growth and primary metabolism, presence of *Protaphorura* induced the expression of defence-related GO terms, suggesting that they primed the defence against herbivores during SF and RF. Notably, Collembola triggered alterations in gene expression patterns and plant allocation in non-mycorrhizal plants, but they also interacted with mycorrhiza in altering plant performance with the effects of both varying markedly between plant growth phases. The results suggest that oaks recognize the presence of Collembola and respond by increasing the allocation of carbon into growth and by preparing against herbivore attacks in particular during SF. In contrast, during RF *Protaphorura* stimulated plant fortification and secondary growth. Overall, the results document that both plant gene expression and allocation patterns can only be understood by considering the multitude of biotic interactors including root associated soil invertebrates. Focusing on soil microarthropods the results document that plant performance and defence gene expression is not only modified by herbivores above and below ground, but also by detritivore animals in soil, highlighting the role of the decomposer system for plant performance and aboveground food webs.

## Supplementary Material

Statistical analysis

## Supplementary Material

Pooling of plants for sequencing

## Supplementary Material

Total Gene Ontology Terms

## Data accessibility

All data needed for replicating this work are provided with this article and the included electronic supplementary material.
