# Supplementary material for: Collembola interact with mycorrhizal fungi in modifying oak morphology, C and N incorporation and transcriptomics
Source: R Soc Open Sci. 2019 Mar 6;6(3):181869. doi: 10.1098/rsos.181869 (PMC6458381; doi:10.1098/rsos.181869)
Supplement: Statistical analysis [file rsos181869supp1.doc]

**Table S1:** GLM table of F- and P-values on the effect of *Protaphorura* (with and without), *Piloderma* (with and without) and Stage [root (RF) and shoot flush (SF)], and their interaction on morphology, ^13^C atom% excess and ^15^N atom% excess of oak microcuttings. Significant effects are given in bold.

| **factor** | **dependent variable** | **df** | **F-value** | **P-value** |
| --- | --- | --- | --- | --- |
| ***Protaphorura*** | leaf area | 1, 70 | 0.83 | 0.367 |
|  | total plant biomass dry weight | 1, 70 | 3.24 | 0.076 |
|  | sink leaves dry weight | 1, 70 | 1.58 | 0.213 |
|  | source leaves dry weight | 1, 66 | 0.41 | 0.523 |
|  | principal roots dry weight | 1, 69 | **4.36** | **0.040** |
|  | lateral roots dry weight | 1, 70 | 2.83 | 0.097 |
|  | stem length | 1, 70 | **4.68** | **0.034** |
|  | relative growth rate | 1, 53 | **5.81** | **0.019** |
|  | sink leaves ^13^C atom% excess | 1, 26 | 2.04 | 0.166 |
|  | source leaves ^13^C atom% excess | 1, 30 | **4.34** | **0.046** |
|  | stem ^13^C atom% excess | 1, 31 | 0.02 | 0.883 |
|  | lateral roots ^13^C atom% excess | 1, 22 | 0.39 | 0.539 |
|  | principal roots ^13^C atom% excess | 1, 29 | <0.01 | 0.981 |
|  | sink leaves ^15^N atom% excess | 1, 26 | 0.36 | 0.553 |
|  | source leaves ^15^N atom% excess | 1, 30 | 0.07 | 0.790 |
|  | stem ^15^N atom% excess | 1, 31 | 0.42 | 0.521 |
|  | lateral roots ^15^N atom% excess | 1, 22 | 0.32 | 0.578 |
|  | principal roots ^15^N atom% excess | 1, 29 | 0.43 | 0.519 |
|  |  |  |  |  |
| ***Piloderma*** | leaf area | 1, 70 | **12.47** | **0.001** |
|  | total plant biomass dry weight | 1, 70 | **6.95** | **0.010** |
|  | sink leaves dry weight | 1, 70 | **7.34** | **0.009** |
|  | source leaves dry weight | 1, 66 | **9.49** | **0.003** |
|  | principal roots dry weight | 1, 69 | 1.12 | 0.294 |
|  | lateral roots dry weight | 1, 70 | **6.94** | **0.010** |
|  | stem length | 1, 70 | **4.17** | **0.045** |
|  | relative growth rate | 1, 53 | **8.60** | **0.005** |
|  | sink leaves ^13^C atom% excess | 1, 26 | **14.45** | **<0.001** |
|  | source leaves ^13^C atom% excess | 1, 30 | 0.08 | 0.782 |
|  | stem ^13^C atom% excess | 1, 31 | **5.20** | **0.030** |
|  | lateral roots ^13^C atom% excess | 1, 22 | 0.25 | 0.620 |
|  | principal roots ^13^C atom% excess | 1, 29 | 2.31 | 0.140 |
|  | sink leaves ^15^N atom% excess | 1, 26 | **12.73** | **0.001** |
|  | source leaves ^15^N atom% excess | 1, 30 | 0.21 | 0.652 |
|  | stem ^15^N atom% excess | 1, 31 | **19.99** | **<0.001** |
|  | lateral roots ^15^N atom% excess | 1, 22 | 0.21 | 0.652 |
|  | principal roots ^15^N atom% excess | 1, 29 | **13.67** | **0.001** |
|  |  |  |  |  |
| **Stage** | leaf area | 1, 70 | 3.42 | 0.069 |
|  | total plant biomass dry weight | 1, 70 | 0.56 | 0.455 |
|  | sink leaves dry weight | 1, 70 | **6.27** | **0.015** |
|  | source leaves dry weight | 1, 66 | **9.14** | **0.004** |
|  | principal roots dry weight | 1, 69 | **7.58** | **0.008** |
|  | lateral roots dry weight | 1, 70 | 0.57 | 0.451 |
|  | stem length | 1, 70 | 2.16 | 0.146 |
|  | relative growth rate | 1, 53 | 0.04 | 0.852 |
|  | sink leaves ^13^C atom% excess | 1, 26 | **53.56** | **<0.001** |
|  | source leaves ^13^C atom% excess | 1, 30 | 3.92 | 0.057 |
|  | stem ^13^C atom% excess | 1, 31 | 0.32 | 0.578 |
|  | lateral roots ^13^C atom% excess | 1, 22 | 2.69 | 0.115 |
|  | principal roots ^13^C atom% excess | 1, 29 | 3.82 | 0.060 |
|  | sink leaves ^15^N atom% excess | 1, 26 | **17.85** | **<0.001** |
|  | source leaves ^15^N atom% excess | 1, 30 | 3.56 | 0.069 |
|  | stem ^15^N atom% excess | 1, 31 | **9.17** | **0.005** |
|  | lateral roots ^15^N atom% excess | 1, 22 | **7.42** | **0.012** |
|  | principal roots ^15^N atom% excess | 1, 29 | 17.06 | **<0.001** |
|  |  |  |  |  |
| ***Protaphorura*** **×** ***Piloderma*** | leaf area | 1, 70 | 1.46 | 0.231 |
|  | total plant biomass dry weight | 1, 70 | 1.21 | 0.276 |
|  | sink leaves dry weight | 1, 70 | 0.03 | 0.863 |
|  | source leaves dry weight | 1, 66 | 3.20 | 0.078 |
|  | principal roots dry weight | 1, 69 | 0.45 | 0.505 |
|  | lateral roots dry weight | 1, 70 | 0.48 | 0.489 |
|  | stem length | 1, 70 | **4.13** | **0.046** |
|  | relative growth rate | 1, 53 | 1.28 | 0.262 |
|  | sink leaves ^13^C atom% excess | 1, 26 | 3.28 | 0.082 |
|  | source leaves ^13^C atom% excess | 1, 30 | 1.33 | 0.258 |
|  | stem ^13^C atom% excess | 1, 31 | <0.01 | 0.973 |
|  | lateral roots ^13^C atom% excess | 1, 22 | 0.11 | 0.748 |
|  | principal roots ^13^C atom% excess | 1, 29 | <0.01 | 0.977 |
|  | sink leaves ^15^N atom% excess | 1, 26 | 0.66 | 0.425 |
|  | source leaves ^15^N atom% excess | 1, 30 | 0.28 | 0.599 |
|  | stem ^15^N atom% excess | 1, 31 | 0.61 | 0.439 |
|  | lateral roots ^15^N atom% excess | 1, 22 | 1.03 | 0.320 |
|  | principal roots ^15^N atom% excess | 1, 29 | 0.42 | 0.522 |
|  |  |  |  |  |
| ***Protaphorura* × Stage** | leaf area | 1, 70 | 0.02 | 0.883 |
|  | total plant biomass dry weight | 1, 70 | 0.85 | 0.360 |
|  | sink leaves dry weight | 1, 70 | 3.17 | 0.079 |
|  | source leaves dry weight | 1, 66 | 1.23 | 0.272 |
|  | principal roots dry weight | 1, 69 | 2.56 | 0.114 |
|  | lateral roots dry weight | 1, 70 | 2.64 | 0.109 |
|  | stem length | 1, 70 | 0.42 | 0.518 |
|  | relative growth rate | 1, 53 | 0.38 | 0.541 |
|  | sink leaves ^13^C atom% excess | 1, 26 | 1.01 | 0.324 |
|  | source leaves ^13^C atom% excess | 1, 30 | **4.82** | **0.036** |
|  | stem ^13^C atom% excess | 1, 31 | **7.58** | **0.010** |
|  | lateral roots ^13^C atom% excess | 1, 22 | 1.01 | 0.326 |
|  | principal roots ^13^C atom% excess | 1, 29 | 0.39 | 0.538 |
|  | sink leaves ^15^N atom% excess | 1, 26 | 3.32 | 0.080 |
|  | source leaves ^15^N atom% excess | 1, 30 | 0.02 | 0.901 |
|  | stem ^15^N atom% excess | 1, 31 | 0.01 | 0.915 |
|  | lateral roots ^15^N atom% excess | 1, 22 | 1.29 | 0.269 |
|  | principal roots ^15^N atom% excess | 1, 29 | **4.73** | **0.038** |
|  |  |  |  |  |
| ***Piloderma* × Stage** | leaf area | 1, 70 | 0.07 | 0.787 |
|  | total plant biomass dry weight | 1, 70 | 0.57 | 0.451 |
|  | sink leaves dry weight | 1, 70 | 3.17 | 0.079 |
|  | source leaves dry weight | 1, 66 | 1.57 | 0.214 |
|  | principal roots dry weight | 1, 69 | 0.13 | 0.720 |
|  | lateral roots dry weight | 1, 70 | 0.64 | 0.428 |
|  | stem length | 1, 70 | 0.02 | 0.888 |
|  | relative growth rate | 1, 53 | 1.83 | 0.182 |
|  | sink leaves ^13^C atom% excess | 1, 26 | **11.88** | **0.002** |
|  | source leaves ^13^C atom% excess | 1, 30 | 0.01 | 0.905 |
|  | stem ^13^C atom% excess | 1, 31 | 0.57 | 0.455 |
|  | lateral roots ^13^C atom% excess | 1, 22 | 2.60 | 0.121 |
|  | principal roots ^13^C atom% excess | 1, 29 | 3.39 | 0.076 |
|  | sink leaves ^15^N atom% excess | 1, 26 | **12.38** | **0.002** |
|  | source leaves ^15^N atom% excess | 1, 30 | 0.06 | 0.803 |
|  | stem ^15^N atom% excess | 1, 31 | 2.86 | 0.101 |
|  | lateral roots ^15^N atom% excess | 1, 22 | 1.48 | 0.237 |
|  | principal roots ^15^N atom% excess | 1, 29 | **10.26** | **0.003** |
|  |  |  |  |  |
| ***Protaphorura* × *Piloderma*× Stage** | leaf area | 1, 70 | 0.20 | 0.659 |
|  | total plant biomass dry weight | 1, 70 | 1.13 | 0.291 |
|  | sink leaves dry weight | 1, 70 | 0.16 | 0.690 |
|  | source leaves dry weight | 1, 66 | 1.93 | 0.169 |
|  | principal roots dry weight | 1, 69 | **4.01** | **0.049** |
|  | lateral roots dry weight | 1, 70 | 0.08 | 0.775 |
|  | stem length | 1, 70 | 0.96 | 0.331 |
|  | relative growth rate | 1, 53 | <0.01 | 0.970 |
|  | sink leaves ^13^C atom% excess | 1, 26 | **4.83** | **0.037** |
|  | source leaves ^13^C atom% excess | 1, 30 | 1.36 | 0.253 |
|  | stem ^13^C atom% excess | 1, 31 | 0.09 | 0.770 |
|  | lateral roots ^13^C atom% excess | 1, 22 | 3.13 | 0.091 |
|  | principal roots ^13^C atom% excess | 1, 29 | <0.01 | 0.992 |
|  | sink leaves ^15^N atom% excess | 1, 26 | **4.31** | **0.048** |
|  | source leaves ^15^N atom% excess | 1, 30 | 2.52 | 0.123 |
|  | stem ^15^N atom% excess | 1, 31 | 1.28 | 0.266 |
|  | lateral roots ^15^N atom% excess | 1, 22 | 1.44 | 0.243 |
|  | principal roots ^15^N atom% excess | 1, 29 | 0.37 | 0.549 |
