# Supplementary material for: Collembola interact with mycorrhizal fungi in modifying oak morphology, C and N incorporation and transcriptomics
Source: R Soc Open Sci. 2019 Mar 6;6(3):181869. doi: 10.1098/rsos.181869 (PMC6458381; doi:10.1098/rsos.181869)
Supplement: Pooling of plants for sequencing [file rsos181869supp2.doc]

**Table S2:** Number of plants pooled for RNA extraction and sequencing, and ^13^C and ^15^N analysis of the three treatments: Stage [root (RF) and shoot flush (SF)], *Protaphorura* [with (+) and without (-)], *Piloderma* [with (+) and without (-)].

|  | Stage | *Protaphorura* | *Piloderma* | No. of plants |
| --- | --- | --- | --- | --- |
| Pool 1 | RF | - | - | 3 |
| Pool 2 | RF | - | - | 3 |
| Pool 3 | RF | - | - | 2 |
| Pool 4 | RF | + | - | 2 |
| Pool 5 | RF | + | - | 2 |
| Pool 6 | RF | - | + | 4 |
| Pool 7 | RF | - | + | 4 |
| Pool 8 | RF | - | + | 4 |
| Pool 9 | RF | + | + | 5 |
| Pool 10 | RF | + | + | 5 |
| Pool 11 | RF | + | + | 4 |
| Pool 12 | RF | + | + | 3 |
| Pool 13 | SF | - | - | 3 |
| Pool 14 | SF | - | - | 3 |
| Pool 15 | SF | - | - | 3 |
| Pool 16 | SF | + | - | 3 |
| Pool 17 | SF | + | - | 3 |
| Pool 18 | SF | - | - | 3 |
| Pool 19 | SF | - | - | 3 |
| Pool 20 | SF | + | + | 3 |
| Pool 21 | SF | + | + | 2 |
